# Tryptophan in Nutrition and Health

**DOI:** 10.3390/ijms23105455

**Published:** 2022-05-13

**Authors:** Burkhard Poeggeler, Sandeep Kumar Singh, Miguel A. Pappolla

**Affiliations:** 1 Johann-Friedrich-Blumenbach-Institute for Zoology and Anthropology, Faculty of Biology and Psychology, Georg-August-University of Göttingen, Am Türmchen 3, 33332 Gütersloh, Germany; 2 Indian Scientific Education and Technology Foundation, Lucknow 226002, India; sandeeps.bhu@gmail.com; 3 Department of Neurology, University of Texas Medical Branch, 301 University Boulevard, Galveston, TX 77555, USA; pappolla@aol.com

Tryptophan is a rate-limiting essential amino acid and a unique building block of peptides and proteins. This largest amino acid serves as the precursor for important endogenous indoleamines such as serotonin, N-acetylserotonin and melatonin, which act as neurotransmitters, neuromodulators and neurohormones. An enhanced synthesis of these signaling molecules can improve health, quality-of-life and well-being. The main metabolic pathway of tryptophan is the oxidation to bioactive kynurenines and niacin. Kynurenic acid is the most potent endogenous anti-exitotoxic agent. Other highly relevant pathways of tryptophan are the reversible transamination to indole-3-pyruvate with the formation of the related indolic acids as well as the synthesis of indole compounds and their derivatives by side chain cleavage.

The indolic acids act as potent protective antioxidant agents, whereas the indoles such as indole, indoxyl and indoxyl sulfate are reactive compounds that are primarily studied because of their potential toxicity. Research on the physiology and pathophysiology of tryptophan metabolism has revealed a key role for the amino acid and its metabolites as endogenous molecular master regulators of physiology and plasticity in development and aging. The ratio of tryptophan to kynurenine is a key parameter reflecting endogenous adaptation to stress determining inflammation and degeneration. Tryptophan metabolites such as melatonin and structurally related agents such as indole-3-propionic acid act as potent catalytic antioxidant and bioenergetic agents that facilitate regeneration and protection against stress and aging.

Several indole compounds act as uremic toxins since these agents can induce radical formation that is associated with enhanced oxidative stress and damage. The exploration of the effects of these protective and toxic tryptophan-derived agents has revealed important molecular mechanisms and mediators of adaptation and aging. Research on tryptophan in nutrition and health can facilitate the development of new approaches to extending human health and lifespan. Amino acids are the building blocks of life that enable repair as well as recycling and regeneration in the body and the brain. Research on nutrients, including amino acids such as tryptophan and its metabolites as well as peptides and proteins, or extracts containing this molecular metabolism’s modifiers can improve health. Research into the indololome is a new emerging and rapidly growing field of utmost relevance to science and society.

The Special Issue on “Tryptophan in Nutrition and Health” reports on the broad field of tryptophan research and has examined the key tryptophan pathways and their molecular targets that mediate the effects of the amino acid and its metabolites on nutrition and health (Figure 1).

The latest developments with the rapid progress in tryptophan research are the focus of this collection of articles, and the studies herein demonstrate the relevance of tryptophan and its metabolites that form the indobolome on nutrition and health. The discovery of a broad range of bioactive compounds derived from tryptophan can enable a better understanding of the unique role of this amino acid in physiology and development. New methods have become available and allow us to establish a biomimetic precision pharmacology to prevent disease and protect health. The complexity of the current activities in exploring the many different effects of tryptophan and its metabolites demonstrates the necessity for new approaches in targeting the physiology and pharmacology of indoleamines, indoles and kynurenines. Bioavailability by food consumption, protein degradation or colonic formation in symbiotic organisms seem to be key issues as tryptophan can induce its own depletion, especially under conditions of stress and disease.

This Special Issue contains seven reviews and nine original research articles that conclusively demonstrate developmental programming and reprogramming [1], uniqueness of tryptophan [2], the role of 5-hydroxytryptophan [3], tryptophan AhR-ligands in the skin [4], the impacts of tryptophan metabolites on coronavirus pathophysiology [5], tryptophan metabolism in organ transplantation [6], gut-derived 5-hydroxytryptamin [7], effects of AhR-ligands on melanoma cells [8], effects of stress and escitalopram on genes of the tryptophan catabolite pathways [9], formation of tryptophan derivatives by *Saccharomyces cerevisiae* [10], the relationship between tryptophan-related fluorescence in urine and malignant melanoma [11], the effects of tryptophan supplementation on milk protein synthesis and energy metabolism [12], the induction of tryptophan deficiency and dysbiosis with associated increased systemic inflammation in aged mice [13], the effects of tyrosine and tryptophan supplementation on diet-induced obesity [14], degradation products of tryptophan [15], and the molecular interactions of nitrofurantoin and albumin [16].

The research not only demonstrates that only a sufficient supply of tryptophan can improve, sustain and maintain health but also indicates that increased oxidative tryptophan degradation can lead to the formation of toxic compounds that have detrimental effects. Tryptophan is a double-edged sword, and interventions that modify its metabolism have to be carefully designed to address the specific needs of the target population. The selective improvement of tryptophan metabolism constitutes a great chance to meet the urgent challenge of chronic diseases associated with premature aging, inflammation and degeneration. The decisive endogenous molecular mechanisms and mediators that affect and determine the effects of tryptophan metabolism are covered by this Special Issue and allow for the development of effective strategies to implement prevention, protection and therapy.

## Figures and Tables

**Figure 1:**
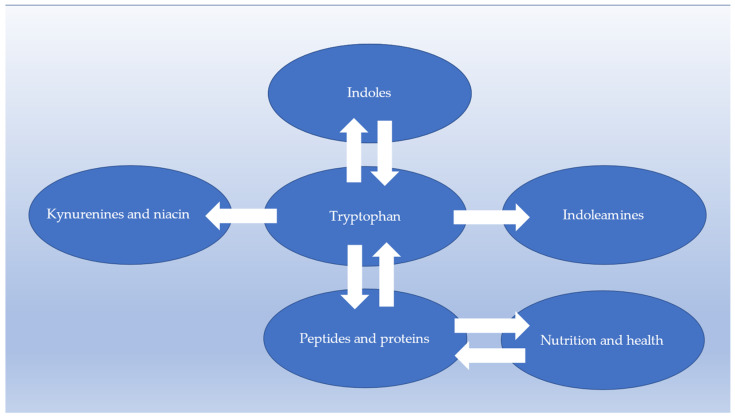
Tryptophan and its main metabolic pathways in nutrition and health.

## Data Availability

Not applicable.
